# Valedictory to the Graduates of Rush Medical College, Feb. 27th, 1861

**Published:** 1861-03

**Authors:** J. Adams Allen

**Affiliations:** Prof. Prin. and Prac. of Med., and Clin. Med.


### ORIGINAL COMMUNICATIONS.

### VALEDICTORY

TO THE GRADUATES OF RUSH MEDICAL COLLEGE, FEB. 27TH, 1861.

BY J. ADAMS ALLEN, A. M., M. D.,

*Prof. Prin. and Prac. of Med., and Clin. Med.*

*Gentlemen of the Graduating Class :*

The pleasant duty of welcoming you to the ranks of our honorable profession has been devolved upon me, and I accept the trust with the greater alacrity, because I am fully convinced that in accepting you as Doctors of Medicine, the Trustees and Faculty of Rush Medical College can, with justifiable and honorable pride, assert that you have worthily won, and we are fully convinced that you will honorably sustain, this respectable character. Custom has sanctioned the usage of commending to the attention of graduates, upon an occasion like the present, certain suggestions of a more general nature than those which we, as your instructors, have previously had opportunity to convey, and yet which we believe will be found practically useful in your future career.

It is unnecessary to remind you that your medical education, so far from being finished, is this day but just commenced —alas, that so many who have gone out from the various schools of medicine so often think otherwise!

You have secured the right to append M. D. to your autograph, but be pleased to remember that there is nothing cabalistic about these mysterious letters—you will yet learn, if you have not already, that these will not of themselves prove an "Open Sesame" before which the gates of success and prosperity will spontaneously swing wide and free for your ingress.

The degree of Doctor of Medicine is, perhaps, a ticket of admission to the race ground of professional life, but your success upon the course, thereafter, must depend upon the kind and vigor of your subsequent efforts.

We fervently trust that in the coming time, the Faculty of this College may have no occasion to say to any one of its athletæ, this day admitted to the friendly contest: "*Ye did run well, what hath hindered you?*"

We regret to inform you that the course, intervening between you and the goal you seek, is not altogether free from obstacles which may seriously impede, or, if you are not prepared to overcome them, altogether check your progress. The track is not a clear one, on the contrary it is beset with snares and pitfalls, hurdles and five-barred gates, by-ways and cross-roads, pleasant to the view, perhaps, but terminating in chaos and dishonor.

Another thing, although the Grecian monarch refused to run the Olympic race unless he could have kings for competitors, you cannot imitate his royal reticency. You will have to make your way, side by side, with the unseemly mounte-bank, decked perhaps with pods of Capsicum, Lobelia stalks, and a steam boiler to make up for his acknowledged light weight. And then next him, "Lo, the poor Indian!" makes the white runner who steals his moccasins and herbs, rich in wings of speed like Mercury (*not* Calomel) of old mythology—the seven leagued boots of nursery tale are nothing to them. And then comes the so-called Eclectic—"a catcher up of unconsidered trifles" thrown away by you and your confreres—he fattens upon them like an ass upon thistles, or an ostrich upon bits of window glass and rusty nails, and waxing fat in popular success, he kicks at you in your tardiness and discomfiture.

And next comes a figure which perplexes us to describe, for it seems neither matter nor incorporeal essence—a kind of starveling imitator of Ariel or tricksy Puck in serio-comedy, essaying to put an immaterial girdle around the world of disease within the forty minutes. It does not seem to walk or run, but is *puffed* along by the side of you—outstripping you, as the butterfly or thistle-down eludes the pursuit of the truant schoolboy.

> *It* is perfumed like a milliner,
> And twixt *its* finger and *its* thumb *it* holds
> A "*potency*," which ever and anon
> *It* gives the nose——
>
> * * * And still *it* smiles and talks.
>
> * * * *
>
> With many holiday and lady terms
> *It passes* you. * * *
> *It* tells you the sovereigns't thing on earth,
> Is *Pulsatilla* for an inward bruise,
> And that it was a great pity, so it was,
> That villainous *Calomel* should be digg'd
> Out of the bowels of the harmless earth,
> Which many a good tall fellow had destroyed
> So wretchedly; and, but for these vile drugs
> *It* would *itself* have been a "*Regular*."

It passes you, whilst embroidered handkerchiefs wave out upon it an atmosphere of Cologne from drawing-room windows, where *vapors* stifle all womanly intelligence—benedictions float out upon it from the shutters of dapper preachers whose—

> "Dear dyspepsia grows a dire disease,"

beneath its toothsome attenuations—titillating, if not the stomach, at all events, the fancy of him who undertakes the cure of souls upon quite other grounds than *similia similibus curantur*.

Let them pass on—all—to what of future fortune the world is full of prophetic assurances.

Be not discouraged at the sight of worthless ignorance or viler imposture, from time to time, securing an apparent advantage over you. It is the same in all pursuits involving the employment of intellect and thought. Why even the high truths of revealed religion are little popular among the masses of mankind. They love Joe Smith rather than St. Paul, and will not receive even truth itself unless, as Milton said, arrayed like a notorious falsehood.

How few there are that can grasp the meaning, and the calm trust of the inspired singer of Israel:

"Whither shall I go from thy spirit? or whither shall I flee from thy presence? If I ascend up into Heaven thou art there: if I make my bed in hell, behold thou art there. If I take the wings of the morning, and dwell in the uttermost parts of the sea; even there shall thy hand lead me, and thy right hand hold me. If I say surely the darkness shall cover me; even the night shall be light about me. Yea, the darkness hideth not from thee; but the night shineth as the day; the darkness and the light are both alike to thee. I will praise thee; for I am fearfully and wonderfully made: marvellous are thy works; and that my soul knoweth right well."

How many, alas, are there who choose rather than this the chamber darkened like their own feeble intellects, and then the senseless mummeries, or worse and more wretched than these, the fatuitous blasphemies of modern spiritualism! The follies and absurdities enacted in the name of Medicine, are but the light and trivial shadows projected from the Egyptian darkness of myriad religious superstitions. Sometimes it would seem that there is a compensatory arrangement of some minds, by which it occurs that clearness and profundity in one direction of thought are balanced by dimness of vision and superficiality in another. This is the only way in which I can account for the presence and prayer of a prominent clergyman of this city the other day, at the public *raree*-show of one of the baldest systems of imposture which the present age, prolific as it is of deception, fraud and villainy, has yet produced. And this very system of imposture is thus endorsed:

> "Though contradicted every day,
> By facts which sophistry itself would stumble o'er;
> And to the very teeth a liar proved,
> Ten thousand times."

You must free yourself from the idea that men only need to see the right and true in order to recognize and follow it. Why it is but a few brief months since the "Universal Yankee" boasted at home, and upon all the continents and seas, that he was a subject of the freest, the happiest, the most prosperous and most perfect government the world ever saw. Universal suffrage and the will of the majority were the panaceas for all political woes. And yet the feasting of the "sovereigns" in November, furnished the "funeral baked meats" which did coldly set forth the marriage tables of a new wedding, without even the judicious formula of a legal divorce from the older tie. And now one half of the country is dancing with joy over its new sensation, as Nero fiddled at the burning of Rome; whilst the other half is dissolving in lamentations, as helpless and still as the marble Niobe of ancient fable, shedding stony tears for its slaughtered children.

Gentlemen—There is no accounting for the phases of human folly, for its ludicrous foibles, its whimsical crimes. In Medicine, as in everything else, you must take the world, as you find it—not to whine over it, but to do something in it—something to make it better; something to make you fitter to stay in it, because more dissimilar to the uneducated (and even some of the educated) rabble which choke up *all* the avenues, leading whether to success, or defeat and ruin.

It is well for you to propose to yourselves, clearly and definitely, what you mean by success in professional life. Indeed everything depends upon your apprehension of this idea—for the life which from one point of view may seem a failure, may really be a splendid triumph, and the reputed success in truth a most ignoble defeat.

Do you propose to yourself the success which riches attest? Then you must conjoin to your medical attainments that knowledge of the details of general business, or speculation indispensable to the making of money. The very best authorities on the subject of money getting, assert that the particular kind of trade or profession in which a man may commence his life has, in reality, little to do with the real process of getting rich. The millionaire, Girard, said that the first thousand dollars he accumulated, cost him more real effort and struggle than all the rest of his vast possessions therewith acquired.

To secure this object you must, in medicine as in other pursuits, simply live within your income, however small it may chance to be, and then invest the surplus under ordinary business rules. It is very rarely, only in cases as exceptional as prizes in a lottery, that medical men acquire wealth merely from the surplus of their professional receipts over their expenditures. To acquire the first hundreds or thousands, you must, in your trade of doctor, please your customers, and rigidly collect your bills, precisely as other tradesmen do. These are all the secrets involved in acquiring the success of wealth. But if you make this the sole object, do not complain if by and by you find that you have failed in securing professional distinction, or in acquiring the riches of profound knowledge—ye cannot serve the God of Medicine and Mammon as joint partners.

Or is your ambition directed toward the securing of popular reputation, the fame of a large practice and the winning of golden opinions from all sorts of men and women? If so, you must adopt a different course. You must, like a political demagogue, adapt yourself to the humors and tastes of all with whom you are thrown in contact—literally becoming all things to all men, and especially all women. Read the advice of Polonius to his son Lærtes and bear his,

> " precepts in thy memory.
> Look thou character. Give thy thoughts no tongue,
> Nor any unproportioned thought his act.
> Be thou familiar, but by no means vulgar.
> The friends thou hast, and their adoption tried,
> Grapple them to thy soul with hooks of steel;
> But do not dull thy palm with entertainment
> Of each new hatch'd unfledg'd comrade. Beware
> Of entrance to a quarrel: but being in,
> Bear it that the opposer may beware of thee.
> Give every man thine ear, but few thy voice:
> Take each man's censure, but reserve thy judgment.
> Costly thy habit as thy purse can buy,
> But not expressed in fancy; rich not gaudy:
> For the apparel oft proclaims the man:
> * * * * * *
> Neither a borrower nor a lender be;
> For loan oft loses both itself and friend;
> And borrowing dulls the edge of husbandry."

If you will have this kind of success, *denounce nothing*—in the cant language of the day, be conservative among conservatives, a radical among radicals, but beware of the pen and printer's ink, lest your heedless expressions unhappily rise up in judgment against you elsewhere. If you are connected with a church—within its folds be the chief of sectaries, but outside of that give utterance to the largest catholicity of views.

In your practice charge the leaders of opinion nothing or a nominal sum for your services, and then make up the deficiency, if possible, from those nonentities in society whose complaints are never noticed or regarded. Secure by indirect influences the appearance of your name in the newspapers as often as possible, and to aid in this laudable course, connect your name if possible with eleemosynary associations, especially where, in consideration of your acceptance of an office, they will not call upon you for contributions, except perhaps of words which cost you nothing. Or seize the opportunity of having your name posted upon the corners of all the streets as "the distinguished lecturer" upon some topic which will not involve controversy, or anything beyond the utterance of glittering generalities. When among the adherents to scientific medicine assume to be the soundest of all true Æsculapians, but do not forget when among any of the disciples of other and strange creeds to be ready to admit your own dissatisfaction with many generally received opinions, and descant as learnedly as you can upon the wonderful benefits which have been derived by legitimate medicine from the engrafting upon its lopped limbs of these varied bastard scions. If you have fluent utterance and you are quite sure your grammar is unimpeachable, be "instant in season and out of season" with your tongue,

"To tickle the maggot born in an empty head,"—

but if you have doubts upon this point avoid the *magpie* as your exemplar, and enthrone the *owl* in its place. You can scarcely conceive the power there is in shrewd silence on occasion, or the immense influence of the wise and oracular look.

When Lord Mansfield was Chief Justice of England, a general officer in the army, one of his friends, came to him in great perplexity, saying that he had got the appointment of Governor of a West India island; which made him very happy until he found that aside from his gubernatorial duties he was also to act as a judge, hearing and deciding causes. As he knew nothing whatever of law, this troubled him exceedingly. Lord Mansfield said to him, "Be of good cheer—take my advice, and you will be reckoned a great judge as well as a great commander-in-chief. Nothing is more easy; only hear both sides patiently—then consider what you think justice requires and decide accordingly. But never give your reasons;—for your judgment will probably be right, but your reasons will certainly be wrong." Two or three years after an appeal came up from a decision of the Governor, based entirely upon the ludicrously absurd reasons he had given for the judgment. It turned out on inquiry that the Governor having acquired much reputation by closely following Lord Mansfield's advice, began to suppose himself really a great lawyer, and that this case carried up was the first in which he had given his reasons, and was the first appealed against. Truly, as Carlyle exclaims, "*Great is silence*," for, we may add, by its potent influence the children of this, and all other generations, prove themselves far wiser than the children of light!

I ought not to forget to add that if you are forced to speak of professional matters, as in explaining the case of a patient to inquiring friends, or more particularly in a court of justice where a large audience is present, you must use only the most ultra technical phrases and terms to convey or conceal your ideas—"words of learned length and thundering sound," whose ponderous proportions and unintelligibility will fall upon the senses of the hearers as having great *specific* gravity. To illustrate, in explaining the influence of improper food in causing indigestion, you can say that: "The concatenation of ineffable self-existence proceeding in a hypostatical duplicate ratio from the primordial concoction of the supermundane essence has caused the bacon and greens to degenerate, and thus has given the poor caitiff—a stomach-ache!" Thus you will often gain more "in a flash, than if your brain pan were an empty hull and every muse tumbled a science in."

By these and similar arts you may secure the success of popular reputation—but when attained, if you find yourself despised by your honorable professional brethren, and poor indeed in scientific attainment, contemptible even to yourself—do not complain of the profession for not awarding you success. According to your faith it has happened unto you. As you have made your bed you must lie in it—albeit upon thorns.

Machiavelli says: "The occasion of every man's good or bad fortune consists in his correspondence and accommodation with the time."—The man must seek to adapt himself to the particular circumstances in which he is placed, and make all their influences bend to the accomplishment of the especial object proposed, but when he has thus proved himself the "architect of his own fortunes," he must not blame the times, or the methods, because the completed edifice does not fill up the proportions of his ideal. If you have struggled and fought for a taste of the apples of Sodom, do not scold because on sinking your teeth beneath the cortex, instead of pulp you find ashes. You will say with Paracelsus:

> "You know my hopes;
> I am assured at length those hopes were vain;
> That truth is just as far from me as ever;
> That I have thrown my life away; that sorrow
> On that account is vain, and further effort
> To mend and patch what's marr'ed beyond repairing,
> As useless: and all this was taught to me
> By the convincing, good, old-fashioned method
> Of force—of sheer compulsion."

You will have labored and toiled for the Midas-power of turning whatever you touched to material gold, or the duller metal of popular applause, and if this satisfies you not, beware of lugubrious complainings at fortune for the calamity, lest you be adorned with the already sprouting *Midas-ears.*

The sacred proverb is: "Though thou shouldest bray a fool in a mortar among wheat with a pestle, yet will not his foolishness depart from him," and the experience of later times is, that should you supply the fool with a *mortar* he will not cease of himself to *bray*.

Notoriety, Gentlemen, is a cheap commodity. Plutarch gravely records in the life of Alcibiades, that he had a magnificent dog, the admiration of all the citizens. One day, this much admired animal appeared in the streets of Athens deprived of his tail—curtailed, in short. The dog-fanciers of all Greece were in mourning over this dire extremity, and wondered at Alcibiades for the act. But the biographer lets posterity into the secret. There was a lull in the politics of the time, and Alcibiades was determined still to be talked about —hence the canine misfortune. Without punning—may we not say "thereby hangs a tale" for the lovers of notoriety even in our times? It is a more expeditious and compendious method than many which now are frequently adopted.

I must caution you here, however, that if you attempt notoriety, you must not adopt the outworn, stale methods. None of our communities would now submit to the sottishness of a Paracelsus, the brusqueness of Abernethy, the coarseness of Jebb or the brutality of a Radcliffe. Tremendous neckcloths, flashy waistcoats and all the assumed eccentricities and absurdities of costume have gone the way of wigs, cocked-hats and gold-headed canes. Neither will the ruse of Jehu-driving, aimless and objectless, to the imminent danger of pedestrians, avail. Neither the frequent call from service at church or crowded assemblages—all that was exploded years ago.

Now-a-days people require more delicate manipulation. *Humbug* must "take any other shape but that"—it must be managed with the most perfect tact and finest artistic taste. This thing is now reduced to a scientific system, and if you *must* attempt to strike that vein, you must use a most delicate scalpel, remembering all the time that you are not carving a goose, but practicing one of the most recondite and perplexing of the fine arts.

Whether the desired result is worth the necessary effort, I will leave it for you to determine—merely suggesting that, rather than be a professional Jeremy Diddler, it would be less trouble for you, and certainly of far more advantage to the world, to blow your brains out entirely before you begin to use them thus.

In the time of William and Mary, in the case of *Buxton versus Mingay*, the case turned upon the point, whether within the meaning of the act of Parliament, a *Surgeon* is an *inferior tradesman.* The Chief-Justice Wilson, says the chronicler, took the liberal side, saying: "I am clearly of opinion the legislature could never intend that a Surgeon is an *inferior* tradesman, or dissolute person, although he may sport without being qualified to kill game." But Judge Bathurst added —"I can never be of opinion that the legislature intended to permit every master of every little mechanic trade to neglect his trade and go hunting. I am of opinion that every tradesman is *inferior* who is not *qualified*—and that is the only line we can draw between inferior and superior." This famous decision was always exceedingly distasteful to our English faculty, although the penalty of want of qualification was only the loss of a chance to shoot hares and foxes and grouse. But the gist of the decision is that, whether surgeon or tradesman, he who is not qualified is, and must continue, *inferior*.

That physician who most perfectly qualifies himself to discharge the high duties of his profession, achieves the truest and only real success. That is the success at which each of you, Gentlemen, and every member of the great fraternity of medicine, should most constantly and assiduously aim. Seek *that* qualification if you would rise above the " inferior tradesman " or mere " dissolute person." Seek that success, and all others will be added to you. This is the " royal road " for which the ancient king inquired in vain. We opine that if we look into this idea a little, we shall find something of more value than the aims, methods and results we have thus far noticed.

Between the good and poor physician the only difference is in their respective knowledge. An easy morality suggests that some particular faculty or tact likewise distinguishes them. Admit this, and even then the difference is but one of knowledge. If the difference is real and positive, then is it a subject of investigation, and of ideas which may be cultivated and perceived. He who tells us that one man may not surmount the barriers which separate him from another, in effect declares that he has faculties which may not be developed: he blasphemes the law of God which impresses the attribute of perpetual development upon the human mind in all its parts. The *divina particula auris* breathed into the nostrils of man at the creation will be felt forever—immortality and infinity of growth wait upon it.

All knowledge is in the line of your profession. It is an idle fallacy that would restrict the province of the physician to the mere mechanism and derangements of the material frame. *That* we recognize as the medium of manifestation of all those high forces whereby it is knit and connected in the harmonious series, from the monad to the Supreme. No man as the physician may say—*Homo sum! nihil humanum a me alienum puto.*

The biographer of the celebrated Dr. John Mason Good tells us that he wished to bring himself under the urgent obligation of a *moral necessity* to attain the greatest possible amount of knowledge; and this feeling conducted him to an eminence of attainment which, in any other field than medicine, would have placed him side by side with the highest names which will go down to immortal fame. The sovereignty of man lies hid in knowledge, as Bacon says, and thus the only true success you may possibly attain is by securing this sovereignty.

The Osiris of Egyptian mythology was likewise known as Serapis the God of Medicine. Osiris is considered as the type of the active, beneficent, generating force of nature and of the elements. He was particularly adored in the sun whose rays vivify and impart warmth to the earth, and who on his return in the spring appears to create anew all organic bodies. Isis, of the same myth, represents the passive force or recipient, and was worshipped as the symbol of the earth or sublunary nature in general. United together, Osiris (or Serapis) and Isis typify the Universal Being, the Soul of Nature, the Pantheus of the Orphic verses.

Osiris having enriched Egypt by his benefits visited other countries, instructing them in agriculture and the arts and sciences. On his return, his brother Typhon, with the assistance of other conspirators, killed him, and, dividing his body into many fragments, scattered them throughout Egypt.

Typhon illustrates the destructive principle, ignorance or error, which is still allied to truth, even as a brother. The whole myth mystically alludes to the introduction and influence of error in the world. But Typhon or error and destruction did not altogether prevail, for Isis still remained alive, who sought earnestly for the dissevered fragments of Osiris, and as each was discovered she enclosed it in a statue of wax representing him, and distributed these effigies throughout the country which living he had blessed by his instruction and labors.

Thus, also, once came Truth into the world, most glorious to be seen, but anon also came Error, than which nothing is more destructive, and with its army of deceivers hewed the body of Truth into innumerable fragments, scattering them to all the winds and continents and seas. Since that time the disciples of Truth have gone like mourning Isis, up and down, collecting the dismembered fragments of truth, and though they have not as yet been able to reconstruct the whole body, they have endeavored to embalm each recognized part in an effigy of amber, that it may be preserved to that coming time when, all the particles having been discovered, they may be re-arranged in the body and framework of life imparting Truth. Isis still mourns her own griefs in the absence of the full protective and beneficent influence of Osiris, while the pestilential breath of Typhon mars the fair banks of the Nile.*

Terra salutiferas herbas eademque nocentes;
Nutrit, et urticæ proxima sæpe rosa est.

In assuming the position of *Doctores Medicinæ*, Gentlemen, I take it that you are at the same time inspired with a more fervid zeal in the search for truth. For how is it possible for Serapis to have escaped when Osiris was torn in pieces? Indeed we are inclined to believe he was mangled not as Osiris the active force of nature, but as Serapis the God of Medicine. The brood of Typhon still remain unchained, and the destructive energies still oppose the healing art, sometimes even under the guise of its devotees. When error comes in its proper habiliments, it is easily detected; but there are modes where it creeps into confidence which are not so readily disclosed. You are fully aware that in this province there must be much patience and groping in the dark—much struggling with the uncertain and untrue, before the light of discovery begins to dawn. Yet "the essence of truth is plainness and brightness, the darkness and confusion are all our own." How often has it happened that some spawn of Typhon has put the workings of ignorance into wild and unintelligible language, undertaking to transform philosophical medicine into a dreaming and ignoble mysticism—*asinus portans mysteria.* The way both of deceivers and deceived, like "the way of the wicked is darkness, and they know not at what they stumble."

The peculiar position of medical science between the present and past is fruitful of difficulty to the investigator of truth.† We rely upon the past for facts and experience, upon

* The elaboration of this simile was suggested by a passage in the prose works of Milton.

† Subjectum illud medicinæ (corpus nimirum humanum) exomnibus quae naturæ procreavit maxime est capax remedii; sed vicissim illud remedium maxime est obnoxium errori. Eadem namque subjecti, subtilitas et varietas, et magnam facultatem prœbet, sic maxime etiam aberrandi facultatem.

the present for observation and advance. You can depend upon neither to the exclusion of the other. It is but an idle calumny which asserts that our science is wedded to the dicta or dogmas of historic times. Yet experience, to be valuable, *must* embrace great cycles of time. The apparent fact of a day, or even of a whole life-time, may be proven by a year, or by the centuries to be untenable and false. The phenomena of astronomy are aptly illustrative of this. The "fixed stars" are now known to move, and you must not be surprised to find the "fixed fact" of your own experience, as you may deem it, prove migratory, in obedience to a higher law than your special senses, or even your intellect, can now appreciate. There are some minds which have more respect for the voices coming up from the depths of an obscure antiquity, than for that which descends from the throne of the Omniscient. And so there are others to which, as Ovid says: *Est quoque cunctarum novitas carissima rerum.* The novelty of a thing is all that is needed to commend it to their belief. Practically such minds brand all the accumulated wisdom of the past as folly. But the old and the new are not like the Manichæan principles ever in conflict, but rather as the Castor and Pollux of Roman myth. Castor or the Past is first removed from the earth, but nevertheless still shares the immortality of Pollux or the Present, by the especial direction of Jupiter himself, who we may well recognize as Jupiter Serapis the God of Medicine.

To the seeing eye objects become visible which escape the superficial observer; to the cultivated perception ideas become familiar that the sciolist may never comprehend. To feel the necessity, and subject ourselves to the sway of influences of an appropriate nature, we have but to truly consider what we owe to our race, to ourselves and to our God. Do we need to enlarge upon this point?

To the medical man it is especially requisite that vivid ideas of the objects and responsibilities of his position should con stantly be presented, that thereby his energies, his perceptions and volitions should be correspondently intensified.

You have passed the years of your pupillage in the offices of your preceptors, and in the halls of the Medical College—you go out hence merely to continue the student life. You are to use what you have thus gained for the double purpose of benefiting your fellow men in the hours of pain and danger, and also for gaining an honorable subsistence for yourselves and those dependent upon you. But it will never do for you to depend upon the interest of this little capital. The years will depreciate it—the world will move past you and your little hoard. You may fancy yourself rich, but the accumulating treasures of the coming time, in the hands of those who will eagerly strive for them, will leave you pauperized in the contrast. There is no standing still in this life of ours—you must move onward or you will steadily float backward. Some of you may have entertained the hallucination that if you have cleverly mastered the details of the text book and of the curriculum of lectures, anything beyond is simply "carrying coals to Newcastle,"—but let me assure you, you might do worse than this, provided you carry better coals than they have in Newcastle. There is no danger. Bostonians tell of one of their wealthy families whose progenitor laid the foundation of an immense fortune by carrying *warming pans* to Cuba. Being at a loss for frieght the worthy gentleman asked the advice of a waggish friend, and credulously took him at his word when he jocosely advised him to this strange cargo. But lo, when the vessel reached Cuba, the warming pans were just the article wanted by the planters for syrup and sugar ladles, (the lids for skimmers,) and the happy shipper returned rejoicing with his hold filled with sweetness. The moral is clear as sunlight—it is the idle man that Nature and Fortune despise, but all the gods help those who try to do something themselves.

Many of you will soon commence the duties of practice for the first time. The chances are that it will come to you slowly and gradually—I could almost desire it to be so for your own sakes. This will give you time to investigate each case as it is presented, with great care and minuteness. Never permit yourself to do otherwise, particularly in the early years of your practice. A conclusion, possibly correct, which you jump at, although it may give you temporary *eclat* with the populace, is, in plain language, a professional injustice to yourself and patient. As a mere matter of policy, you had better take the character of the earnest, pains-taking, careful interrogator in every case. I once heard a gentleman, whose name ranks among the highest for legal fame and success, say that he never undertook a case, however apparently clear or trivial, without devoting to it all the labor and thought of which he was capable, as though the reputation of his life depended upon that single case. The result was a continuous success almost without parallel. Take this course with your first patients, young Gentlemen, and your success is certain. You will become thoroughly grounded, profound, expert and strong, when your well earned reputation shall have widened the circle of your practice to its largest limits.

Another thing--now while you have time carefully record each case, with all its characteristics, not merely its *peculiarities*, as is too often done. You will thus cultivate exactness and definiteness in your ideas, and will presently be the possessor of a medical treasury invaluable to you and the world. Adopt some good system, and religiously set down the facts without concealing your mistakes or blunders. We often profit more from unexpected results and blunders than could have been anticipated. The unexpected result will awaken inquiry, and the recognized blunder will forever prevent its own repetition. It will do you a world of good by and by, and encourage you to continue by the evidences of improvement it will daily and monthly exhibit.

But you must not depend upon your own experience alone, but seek to profit by that of all intelligent members of the profession. This is to be done to a limited extent by ordinary social conversation with your colleagues, or more rarely in such well ordered medical societies as you may find which may make it their object to develop the science and art of medicine. The trouble with these is that, owing to the ordinary weaknesses of human nature, they are too often prostituted to the purposes of individual notoriety, the advancement of clannish or clique interests, and especially to "operate" as executioners under the "code."

A better method than this is to purchase regularly such volumes as constitute a staunch evidence of advance in medicine. From the large number of medical periodicals select not less than two for permanent subscription. Ten or fifteen dollars a year for medical periodicals and from twenty-five to fifty per year for medical books ought to be set apart as the least possible sum to be devoted to this purpose. It will pay in reputation although you should never read them; and it will prove a perennial fountain of satisfaction within you, provided you "read, mark, learn and inwardly digest" them.

Knowledge to-day is a cosmopolite. Read the books and journals carefully and conscientiously, and you will have no real need of going to New York, Philadelphia, Edinburgh, Paris, or any other place, in order to find what the medical world is doing or whereof it is thinking. A single trip to the seaboard to see a few operations performed or prescriptions made, either better seen at home, will cost far more than the central suns of a library which will dispense with all further necessity for such smoky torches as those. And here, at the risk of being tedious, allow me to recommend that you carefully *index* all subjects treated upon in the journals or monographs you read. Keep an "account current" with the medical, as you would with the financial, world. Literally, keep "posted up." Then, at a moment's glance, you can tell where to refer for inquiry when any subject comes up for investigation, either for immediate practical use, or for writing upon it. Let nothing escape you. Little by little, the results will accumulate until, when a few years have passed over, you will have a *thesaurus* valuable as a mine of gold. If you propose to do anything in this world of ours, the only way given under heaven among men is to work for it, and the laborer will get his hire. For Heaven's sake do not entertain the sluggard or niggard's view of these ideas—they are immediately and intensely practical.

But if you will not continue studying and reading, be manly about it; don't *sneak* and say you "have no time." The most busily employed men of the profession always find time to read, and, what is more, to write. When I hear a physician say that he has no time to read, I am very apt to set down that man as a billiard or card-player; or, worse than either, a politician. The chances are that he will follow an ill looking dog, and carry a rifle and knapsack, for miles and miles, over logs and through thickets and brush heaps to get a chance to shoot at and miss a chipmunk or a crow. Or you will find him on the street corners, more anxious than the men of Athens "to hear and to tell some new thing" about anything else than medicine. I pray you avoid these things.

You must not expect that there will be a sensation created when you (prepared as well even as you are,) go out into the business world.

> "You'll do some excellent things indifferently,
> Some bad things excellently. Both be praised,
> The latter loudest."

Things will move on pretty much as before. You may have a good hold of the Archimedean lever, but it will be some little time before you will get a place to stand, and alas, infinitely longer still before you will find a fulcrum by means of all which you may reasonably expect to move the world. *Festina lente*—your influence will be felt although you can scarce appreciate the result, for

> "It must oft fall out
> That one whose labor perfects any work
> Shall rise from it with eye so worn, that he
> Of all men least can measure the extent
> Of what he has accomplished. He alone
> Who, nothing tasked, is nothing weary too,
> Can clearly scan the little he effects."

You cannot depend upon your genius, your tact, or anything whatever except solid attainment, for real and substantial success. When you happen to succeed, irrespective of these, do not be puffed up, but recollect how the Egyptian magicians, with their adroit enchantments, imitated Moses. The ass in a lion's skin is not a commendable or admirable quadruped. Remember that the only thing which makes the so-called liberal professions differ from mere handicrafts, is the amount of brains worked into them. Rather than be a brainless Doctor you had better be a street or chimney sweeper.

The course of instruction which this evening terminates, it is pleasurable in the highest degree to record, has been unclouded by anything which, so far as we know, could mar our mutual satisfaction. The hours have been filled to overflowing with their recurring duties, and whilst as your temporary instructors we have spared no pains or effort to make your attendance here both pleasant and profitable, we should do injustice to our feelings did we not gratefully acknowledge that upon your part, Gentlemen, there has ever been manifest an appreciative attention, a punctuality and promptness, a studiousness of habit and courtesy of demeanor which, in an unusual degree, elicit our sincerest thanks, and personally link you to our hearts in ties of warmest friendship. We leave the conventionally reserved and almost cold relation of instructor and pupil, and meet you upon the level of individual feeling. I am positive that I do not unwarrantably commit my colleagues when I assure each of you, Gentlemen, that all along the diverging paths of your several lives, the earnest good wishes and fervent God-speed of the Faculty of old Rush Medical College will ever attend you.

It seems trite and common-place to say we shall not meet again, but the heart will throb heavier with this deep emotion—

> "Thou shalt hear the "never, never," whispered by the phantom years,
> As a song from out the distance ever ringing in thine ears."

We part, but ever shall our memories, our hopes and aspirations commingle—

> "Through all the bristling fence of nights and days
> Which hedges time in from the eternities."

Pauseless as the pulses of life, we trust your onward and upward progress will keep step to the rhythm of its ever recurring duties.

> "A poor man served by thee shall make thee rich;
> A sick man helped by thee, shall make thee strong;
> Thou shalt be saved thyself by every sense
> Of services which thou renderest."

Gentlemen—My Friends, I waste language in the endeavor to avoid the final word which must be spoken. In the coming battle of life—quit you like men. We have this day given you the well earned guerdon of professional study, and now we bid you our heartfelt—FAREWELL.

